# A molecular network-based pharmacological study on the protective effect of *Panax notoginseng* rhizomes against renal ischemia–reperfusion injury

**DOI:** 10.3389/fphar.2023.1134408

**Published:** 2023-04-18

**Authors:** Dan-Dan Li, Na Li, Chui Cai, Chun-Mian Wei, Guang-Hua Liu, Ting-Hua Wang, Fu-Rong Xu

**Affiliations:** ^1^ Yunnan Key Laboratory of Dai and Yi Medicine, Yunnan University of Chinese Medicine, Kunming, Yunnan, China; ^2^ Department of Laboratory Animal Science, Kunming Medical University, Kunming, Yunnan, China

**Keywords:** *Panax notoginseng* rhizomes, renal ischemia–reperfusion injury, network pharmacology, apoptosis, inflammatory response

## Abstract

**Objective:** We aimed to explore the protective effect of *Panax notoginseng* rhizomes (PNR) on renal ischemia and reperfusion injury (RIRI) and the underlying molecular network mechanism based on network pharmacology and combined systemic experimental validation.

**Methods:** A bilateral RIRI model was established, and Cr, SCr, and BUN levels were detected. Then, the PNR was pretreated 1 week before the RIRI model was prepared. To determine the effects of the PNR in RIRI, histopathological damage and the effect of PNRs to the kidney was assessed, using TTC, HE, and TUNEL staining. Furthermore, the underlying network pharmacology mechanism was detected by screening drug–disease intersection targets from PPI protein interactions and GO and KEGG analysis, and the hub genes were screened for molecular docking based on the Degree value. Finally, the expression of hub genes in kidney tissues was verified by qPCR, and the protein expression of related genes was further detected by Western blot (WB).

**Results:** PNR pretreatment could effectively increase Cr level, decrease SCr and BUN levels, reduce renal infarct areas and renal tubular cell injury areas, and inhibit renal cell apoptosis. By using network pharmacology combined with bioinformatics, we screened co-targets both *Panax notoginseng* (Sanchi) and RIRI, acquired ten hub genes, and successfully performed molecular docking. Of these, pretreatment with the PNR reduced the mRNA levels of IL6 and MMP9 at postoperative day 1 and TP53 at postoperative day 7, and the protein expression of MMP9 at postoperative day 1 in IRI rats. These results showed that the PNR could decrease kidney pathological injury in IRI rats and inhibit apoptotic reaction and cell inflammation so as to improve renal injury effectively, and the core network mechanism is involved in the inhibition of MMP9, TP53, and IL-6.

**Conclusion:** The PNR has a marked protective effect for RIRI, and the underlying mechanism is involved in inhibiting the expression of MMP9, TP53, and IL-6. This striking discovery not only provides fruitful evidence for the protective effect of the PNR in RIRI rats but also provides a novel mechanic explanation.

## 1 Introduction

Renal ischemia and reperfusion injury is the main clinical cause of acute kidney injury (AKI) in critical illnesses (Vanmassenhove et al., 2017), and is also unavoidable in approximately 80% of patients after renal transplantation (Wang et al., 2020b). It is estimated that the annual morbidity and mortality of AKI is increasing worldwide, and most patients are concentrated in developing countries (Mehta et al., 2015). Among them, China had become the region with the most severe incidence of AKI (Zhao and Yang, 2018). Undoubtedly, AKI leads to an increased risk of chronic kidney disease and end-stage renal disease (Chawla et al., 2014; Sato et al., 2020), which are a burden not only for the individual but also for the society, the nation, and the world. Hence, the treatment of AKI is particularly urgent, but there are no effective therapeutic drugs available, leading to the fact that prevention of RIRI is the key to treating kidney injury (Ding et al., 2021), especially since early prevention can improve its prognosis. Previously, the use of pharmacological interventions to reduce ischemia–reperfusion on kidney injury had great clinical importance. Ischemia–reperfusion injury (IRI) can not only induce a pathological condition that initially restricts blood supply to organs and subsequently restores it but also results in blood perfusion and an accompanying imbalance in local tissue oxygen supply and demand, further damaging tissues and organs. Renal ischemic injury occurs mainly in tubular epithelial cells (TECs) and is characterized by ischemic necrosis of TECs as a central feature. In this process, ischemia and hypoxia usually lead to metabolic dysfunction of tissues and organs, causing organismal damage. The specific mechanism of RIRI is not completely clear, but is closely related to apoptosis (Wu et al., 2018), oxidative stress (Han and Lee, 2019), and inflammatory response (Bonavia and Singbartl, 2018; Singbartl et al., 2019).

According to clinical manifestations, RIRI is caused by deficiency of Qi and blood and blood stasis syndrome according to the traditional Chinese medicine (TCM). Therefore, for treating RIRI, TCM can advantageously focus on activating blood circulation, removing blood stasis, and tonifying Qi (Liu et al., 2021b). Of these, the efficacy of *Panax notoginseng* (Sanchi) is appropriate in dispersing blood stasis and stopping bleeding, invigorating blood, and relieving pain. The dried roots and rhizomes of *Panax notoginseng* (Burk.) F.H. Chen, Sanchi mainly contain *Panax notoginseng* saponins (PNS), flavonoids, amino acids, polysaccharides, and other active ingredients; it could be considered a potential indicator for treating AKI. Additionally, Sanchi has modern pharmacological effects such as immune regulation, anticoagulation, antioxidant, free radical scavenging, estrogen-like, anti-inflammatory, vasodilatory, and protection of ischemic–reperfused tissues (Chan et al., 2002; Liu et al., 2020b; Zhang et al., 2021a). Therefore, clinical applications have expanded from traditional cardiovascular and cerebrovascular diseases to also have good therapeutic effects on the kidney, lung, liver, and other parts affected the disease (Zhang et al., 2018; Xie et al., 2020; Liu et al., 2021c; Huang et al., 2022).

Currently, experimental studies have shown that PNS has protective effects on cerebral and myocardial ischemia–reperfusion organs (Wang et al., 2020a; Zhang et al., 2021a), and the effect of Notoginsenoside R_1_ reducing IRI in the kidney is markedly observed (Fan et al., 2020). However, the content of PNS in different parts is different, with the highest content in its rhizomes and the second highest in its roots (Wang et al., 2014). Moreover, as Chinese herbal medicine has multi-component, multi-target, and multi-pathway characteristics, it needs a systematical method to analyze the protective effect of Sanchi on RIRI. Here, by using network pharmacology combined with biological information, we detect the effect of PNR for treating AKI and explore the involved network mechanism. Finally, experimental animal models were designed to demonstrate the protective effect of PNR pretreatment on RIRI. Our data provide a basic theoretical basis for application of Sanchi to improve the treatment of AKI in future clinical practice.

## 2 Materials and methods

### 2.1 Drugs and animals

Drugs: Sanchi (rhizomes, Yunnan Wenshan Kunqi Pharmaceutical Co., Ltd., production batch number: 20211001).

Animals: SPF male Sprague–Dawley rats (weight 200–240 g) provided by the Department of Laboratory Animal Science, Kunming Medical University. No. SCXK (Yunnan) K2020-0004, Animal Ethics Code: kmmu20221856.

### 2.2 Pharmacological experimental design

The bilateral renal ischemia–reperfusion injury model is considered to better simulate the pathological process of AKI in humans and more closely resembles bilateral kidney injury in clinical patients (Wei and Dong, 2012), so the bilateral RIRI model was chosen for this experiment.

SD rats were randomly divided into the sham group (sham, N = 15), model group (IRI, N = 30), and PNR group (PNR, N = 30). According to the clinical dose–effect relationship (equivalent dose ratio of 200-g rats to 70-kg humans converted by body surface area 0.018), the PNR group was administered 270 mg/kg PNR by gavage once daily for 7 days. An equal volume of saline was given to the sham group and the IRI group.

The injury peaked after 24 h of reperfusion and reached a plateau after 7 days, so the mold was taken at days 1 and 7 after successful modeling, and blood, 24 h urine, and both kidneys were collected before the end of the experiment.

### 2.3 Animal models

According to the methods of Yin et al. (2017), the rats were anesthetized with sodium pentobarbital (40 mg/kg) by intraperitoneal injection, 1 h after the last dose, (fasting 12 h before surgery and normal supply of drinking water) and fixed prone on a surgical plate (placed on a heating pad at 37°C–38°C to maintain a constant body temperature). The skin on the back was used, disinfected with iodine, and a longitudinal incision of approximately 2-cm in length was made by cutting the skin layer at the midline of two points under the bilateral rib arches on the back. Then, the incision was pulled above the positions of the left and right kidneys. Afterward, the muscle layer was cut, the bilateral kidneys were bluntly separated and exposed, the bilateral renal arteries were separated, and the bilateral renal arteries were ligated in all the groups of rats, except for the sham group. The kidneys were infiltrated with saline drip at 37°C inside the incision and then covered with sterile gauze to maintain moisture. After ligating the bilateral renal arteries for 40 min and releasing them, the color of the kidney gradually changed from purple-black to bright red, indicating that the kidney was well-perfused again. The heating pad was removed after the rats were awake.

The success of the model was judged by observing the color of the kidney, which changed from red to purple-black in ischemia and from purple-black to red in reperfusion, combined with the analyses of urine and blood biochemistry.

### 2.4 Testing methods

#### 2.4.1 Urine and blood biochemical analysis

The urine at postoperative days 1 and 7 was collected using a metabolic cage and sent to Chenggong Hospital of Kunming Medical University for testing the levels of urinary creatinine (Cr).

At the end of the experiment, blood was collected from the heart, and non-anticoagulated blood samples were prepared and stored at 4°C. The non-anticoagulated blood samples were centrifuged at 4°C and 3 000 r/min for 15 min, and the supernatant (serum) was extracted, and serum creatinine (SCr) and blood urea nitrogen (BUN) levels were measured using an automatic biochemical analyzer (Beckman Coulter K.K.).

#### 2.4.2 Histopathology

##### 2.4.2.1 2, 3, 5-Triphenyl tetrazolium chloride (TTC) staining

After the SD rats were anesthetized, both the kidneys were removed *in vivo*, the blood was quickly dried on the surface and placed in a refrigerator at −80°C for about 20 min, and then removed to cut coronally (thickness about 2 mm) at −20°C. The TTC staining solution was quickly added and placed in a thermostat at 37°C for 30 min to avoid light staining, and then the TTC staining solution was carefully aspirated and added to an appropriate amount of 4% paraformaldehyde, stored at 4°C, and fixed overnight. Then, the sections were taken out and observed and photographed.

##### 2.4.2.2 Hematoxylin and eosin (HE) staining

The kidney tissues were fixed by soaking in 4% paraformaldehyde and coronally cut into two longitudinal flaps to make paraffin sections (thickness about 5 μm), and after dewaxing and hydration, the sections were stained with hematoxylin–eosin stain and sealed, and photographed using a full section scanner (3DHISTECH Ltd.).

##### 2.4.2.3 TdT-mediated dUTP-FITC nick end-labeling (TUNEL) staining

After dewaxing, hydration, and antigen repair, the paraffin sections of kidney tissues at postoperative day 7 were rinsed with 0.01 mol/L PBS (pH = 7.6) thrice, 5 min each time and sealed at room temperature for 3 h by adding an appropriate amount of 0.3% Triton X-100 solution (5% sheep serum preparation). According to the TUNEL kit’s (Roche) instructions, the TUNEL reaction mixture (TdT: fluorescein labeled dUTP = 1 : 9) was added to the cartridge and incubated at 37 °C for 1 h, then stained with DAPI staining solution (DAPI: anti-fluorescence quencher = 1:3000) at room temperature for 5 min, and sealed. The sections was observed and photographed for analysis using a Leica fluorescence microscope (Leica Microsystems Ltd.).

### 2.5 Network pharmacology

#### 2.5.1 Screening of drug ingredients and targets

The constituents and related targets of Sanchi were searched by using the TCMSP database (https://old.tcmsp-e.com/index.php). First, we selected “Herb name” in the search box; entered the drug name; clicked “Search”; set “OB” ≥ 30%, “DL” ≥ 0.18, and other parameters as default values as the screening conditions; and obtained the relevant active ingredients of the drug (Zhao et al., 2021). Then, according to the Molecular ID of the active ingredient, the target protein name corresponding to the active ingredient was queried in Related Targets, the clinically validated targets of the species “*Homo sapiens*” were screened by the UniProt platform (https://www.uniprot.org/), the obtained target names were standardized and corrected by comparing the protein names, the target gene names and corresponding numbers were summarized, and the duplicate and mismatched targets were removed to summarize the potential targets.

#### 2.5.2 Disease gene collection

We searched the GeneCards database (https://www.genecards.org/) and entered the keyword “Renal ischemia–reperfusion injury” to download the gene for RIRI.

#### 2.5.3 Venn intersection diagram

Using the Venny 2.1.0 online platform (https://bioinfogp.cnb.csic.es/tools/venny/), we entered “Drug” in List 1 and “Disease” in List 2, then pasted the corresponding genes to screen 106 “drug–disease intersection targets,” and made the Venn intersection diagram.

#### 2.5.4 GO enrichment and KEGG pathway analysis

Through the Metascape platform (https://metascape.org/gp/index.html#/main/step1), we first pasted “Drug–disease intersection target” from Venny to or paste a gene list box, followed by selecting *Homo sapiens* as the species of input, then clicked “Custom analysis,” and finally analyzed BP, CC, MF, and KEGG under “Enrichment” and downloaded the compressed package under “Analysis report page.”

#### 2.5.5 Construction of the protein–protein interaction (PPI) network

According to the drug–disease intersection target in Venny, we opened the STRING network database platform (https://cn.string-db.org/), selected Multiple proteins, then pasted the intersection target in List of name, and selected *Homo sapiens* in Organisms to construct the PPI network map.

#### 2.5.6 Hub gene screening

Hub genes were screened with Cytoscape 3.8.0 software, and the protein interaction table obtained in PPI was imported into Cytoscape. Furthermore, ten hub genes were screened by using the cytoHubba plug-in and Degree value, and histograms were made with the magnitude of the Degree value.

#### 2.5.7 Network relationship diagram of “disease–active drug ingredient–key target–KEGG signaling pathway”

Tables with disease genes, drug active ingredient Mol numbers and their genes, and KEGG signaling pathways were imported into the Cytoscape software to create their network relationship diagram.

#### 2.5.8 Molecular docking analysis

The PDB database was used to download the hub gene protein structure, and the PubChem database was used to download the 2D structure of the drug component. The Open Babel software was used to convert the sdf format of the drug component structure to mol2 format and then to dehydrate and de-ligand it with the PyMOL software. With the gene target protein as the receptor and drug as the ligand, molecular docking analysis was performed with AutoDock Vina software. Finally, PyMOL software was used to embellish it and calculate the hydrogen bond length, label the residue name, and export the ligand, overall picture.

### 2.6 Quantitative real-time (qPCR)

We explored the molecular mechanism of PNR pretreatment on RIRI based on the Hub gene screened by network pharmacology and molecular docking results. An appropriate amount of rat kidney tissues on postoperative days 1 and 7 was selected, homogenized with 1 ml TRIzol (Takara), and 200 μl chloroform was added to extract the upper aqueous phase. Then, isopropyl alcohol was added for centrifugation, and the RNA was precipitated at the bottom of the tube. The RNA precipitates were washed with 80% ice-cold ethanol treated with DEPC water (Biosharp), and the RNA purity and concentration were measured using enzyme marker (Thermo). The reverse transcription reaction system was prepared according to the reverse transcription kit’s (DBI Bioscience) instructions, and the cDNA was placed at −20°C after synthesis and centrifugation. The qPCR reaction system was prepared and gradually added to the 96-well plate. After the sample addition was completed, the reaction was amplified in a PCR instrument (BIO-RAD). The reaction conditions were as follows: 95°C for 5 min, 95°C for 10 s, 55°C–60°C for 20 s, and 72°C for 20 s for a total of 40 cycles. Relative expression was calculated by the 2^−ΔΔCt^ method. The specific primer sequence is shown in Table 1.

**TABLE 1:** Primer sequences.

Gene	Forward	Reverse
AKT1	CATGAACGAGTTTGAGTACCT	CTCCTTCTTGAGGATCTTCAT
TNF-α	CCACCACGCTCTTCTGTC	GCTACGGGCTTGTCACTC
IL6	GCCTTCCCTACTTCACAAGT	GCCATTGCACAACTCTTTTCT
IL1B	GAGCTGAAAGCTCTCCACCT	TTCCATCTTCTTCTTTGGGT
TP53	CCTTACCATCATCACGCTGGAAGAC	AGGACAGGCACAAACACGAACC
VEGFA	CTTCAAGCCGTCCTGTGTG	GCTCATCTCTCCTATGTGCT
CASP3	CGGGTCATGGTTCATCCAGT	CTCAAATTCCGTGGCCACCT
PTGS2	ACTCTATCACTGGCATCCG	GAGCAAGTCCGTGTTCAAG
MMP9	CCTCAAGTGGCACCATCAT	GCGACACCAAACTGGATGA
HIF1A	TCCCATACAAGGCAGCA	GAAACCCCACAGACAACAA
GAPDH	GACATGCCGCCTGGAGAAAC	AGCCCAGGATGCCCTTTAGT

### 2.7 Western blot

Proteins were extracted from kidney tissues using RIPA lysis buffer and centrifuged at 4°C and 12,000 rpm for 15 min, and the supernatant was extracted for use. The protein concentration was determined by a BCA kit (Beyotime). After separation using 12.5% SDS-PAGE gels (EpiZyme), proteins were transferred to PVDF membranes and the membranes were sealed in 5% skim milk for 1 h. Then, the membranes were incubated with primary antibody 3²-actin (1:5000, mouse antibody, 43 KD, Affinity), MMP9 (1:1000, rabbit antibody, 78 KD, Affinity) in a vertical shaker at 4°C for 16 h. After rinsing, sheep anti-rabbit IgG or sheep anti-mouse IgG (1: 5,000, Abbkine) was added and incubated for 1 h at room temperature. Finally, an ECL chemiluminescence substrate kit (Biosharp) was used for development, image information was acquired in the gel imager, and the data were processed using ImageJ software.

### 2.8 Statistical analysis

One-way ANOVA and statistical comparison among groups were performed using SPSS 26.0 software, the LSD test was applied when variance was homogeneous, Dunnett’s T3 test was applied when the variance of data in qPCR was uneven, and the Tamheri test was applied when the variance of other experimental data was uneven. *P* < 0.05 was considered significant, and GraphPad Prism 8.0 was used to indicate significance between the two groups.

## 3 Results

### 3.1 PNR improves renal injury after IRI

**FIGURE 1:**
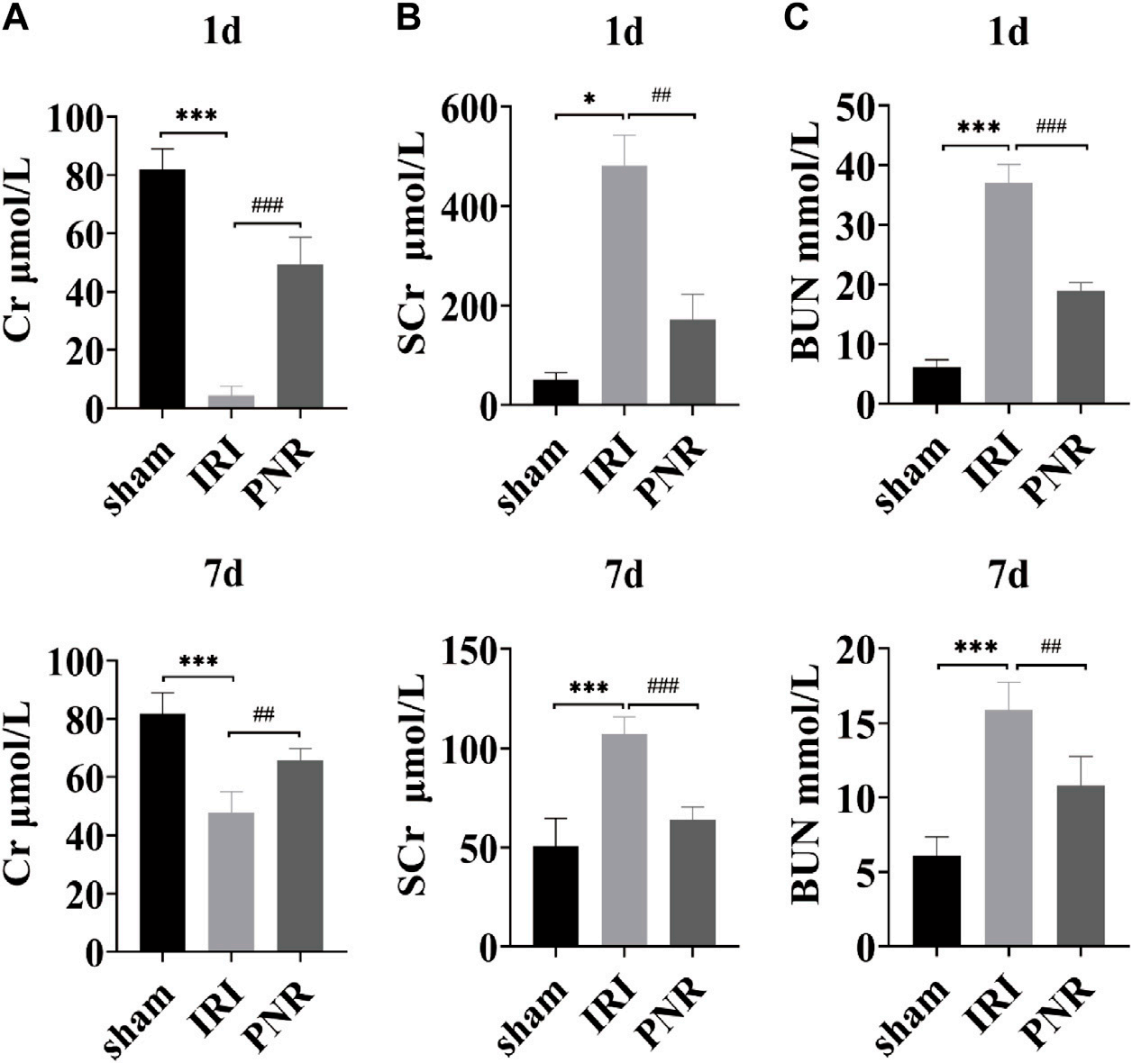
Results of the effect of PNR pretreatment on urine and blood biochemical indexes at days 1 and 7 postoperatively. **(A)** Cr content in each group (N = 4–6); **(B)** SCr content in each group (N = 3–7); **(C)** BUN content in each group (N = 4–7). Compared with the sham group, ***p < 0.001, *p < 0.05; compared with the IRI group, ^###^ *p* < 0.001, ^##^ *p* < 0.01.

Creatinine and blood urea nitrogen were used as common biomarkers for early kidney injury. Compared with the sham group, the IRI group showed significant decrease in the Cr content and increase in the SCr and BUN content at postoperative days 1 and 7, confirming a decrease in glomerular filtration rate and renal dysfunction, and the changes in each index at postoperative day 1 were higher than those at day 7, indicating greater renal damage at postoperative day 1. Moreover, compared with the IRI group, the Cr level was significantly higher and the SCr and BUN levels were significantly lower in the PNR group, both on postoperative days and 7, and the results of each index showed that the effect of pharmacological intervention was better for postoperative day 1 than for day 7, which significantly improved the renal injury after RIRI. (Figures 1A–C).

### 3.2 PNR reduced the pathological damage of kidney tissue

**FIGURE 2:**
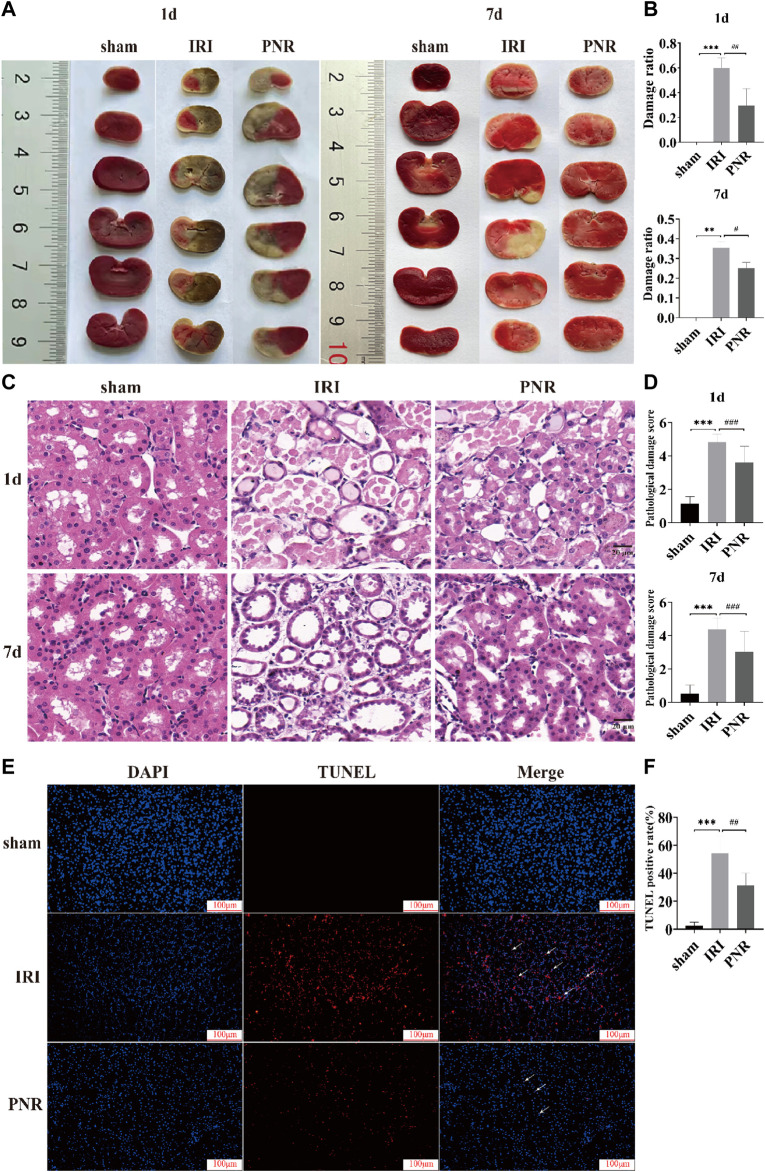
Results of histopathological damage to RIRI kidneys by **pretreatment** with the PNR. **(A)** TTC staining results of PNR pretreatment on each group at postoperative days 1 and 7; **(B)** statistical results of the ratio of TTC staining damage (N = 3–4); **(C)** HE staining results of PNR pretreatment on each group at postoperative days and 7 (×400 magnification, scale bar = 20 μm); **(D)** statistical results of HE staining pathological damage score (N = 3); **(E)** TUNEL staining results of PNR pretreatment on each group at postoperative day 7 (×200 magnification, scale bar = 100 μm); **(F)** statistical results of TUNEL staining-positive cells (N = 3). Compared with the sham group, ***p < 0.001, **p < 0.01; compared with the IRI group, ^###^ *p* < 0.001, ^##^ *p* < 0.01, and ^#^ *p* < 0.05.

The area of tissue infarction was observed by TTC staining of kidney sections, and the sham group was red and had no infarcted area as observed by the staining results. Compared with the sham group, the IRI group clearly showed non-red areas, indicating tissue infarction and an increase in the area of tissue damage resulting from postoperative day 1 compared with day 7. In contrast, compared with the IRI group, the PNR group had significantly fewer non-red areas, less infarct area, and significantly less damage at postoperative day 7 compared with day 1. (Figures 2A, B).

HE staining was used to observe the TEC morphology and score pathological damage such as swelling, vacuolization, and tubular formation of TECs with reference to LIU et al. (Liu et al., 2020a). A total of ten fields of view were randomly selected and scored 0–5 for TEC pathological injury. The details are as follows: 0: normal; 1: histological changes <10% of the damaged area; 2: similar changes >10% but <25% of the damaged area; 3: similar changes involving >25% but <50% of the damaged area; 4: similar changes involving >50% but <75% of the damaged area; 5: similar changes involving >75% of the damaged area. The results showed that the renal tubular cells in the sham group had normal morphology and tight arrangement. Compared with the sham group, the IRI group had a severe absence of cell morphology at postoperative day 1 and showed tubular formation and protein accumulation in the tubular lumen (*p* < 0.001), and a more slight absence of cell morphology at postoperative day 7, but showed tubular interstitial inflammatory factor infiltration and tubular dilatation (*p* < 0.001). While compared with the IRI group, cell morphology recovered in the PNR group at postoperative day 1, which significantly reduced tubular formation and protein accumulation in the lumen (*p* < 0.001). Cell morphology recovered significantly at day 7 postoperatively, which improved interstitial inflammatory factor infiltration and expansion (*p* < 0.001). Also, it can be seen that the IRI and PNR groups were more severely injured at postoperative day 1 than at day 7 (Figures 2C, D).

TUNEL staining was used to assess the consequence of RIRI and the protective influence of the PNR, and the number of positive cells and apoptosis rate from three random fields were calculated (apoptosis rate = number of positive cells/total number of cells × 100%). The results showed that the sham group had a higher number of DAPI-stained cells, normal cell morphology with sub-circular shape, and almost no apoptosis. Compared with the sham group, the IRI group at postoperative day 7 showed a significant decrease in the number of cells and an increase in the number of positive cells, observed with an irregular change in cell morphology (*p* < 0.001). Compared with the IRI group, the number of cells in the PNR group increased, the cell morphology tended to be normal, and the number of positive cells was significantly reduced (*p* < 0.01) (Figures 2E, F).

### 3.3 Network relationship between Sanchi and RIRI

**FIGURE 3:**
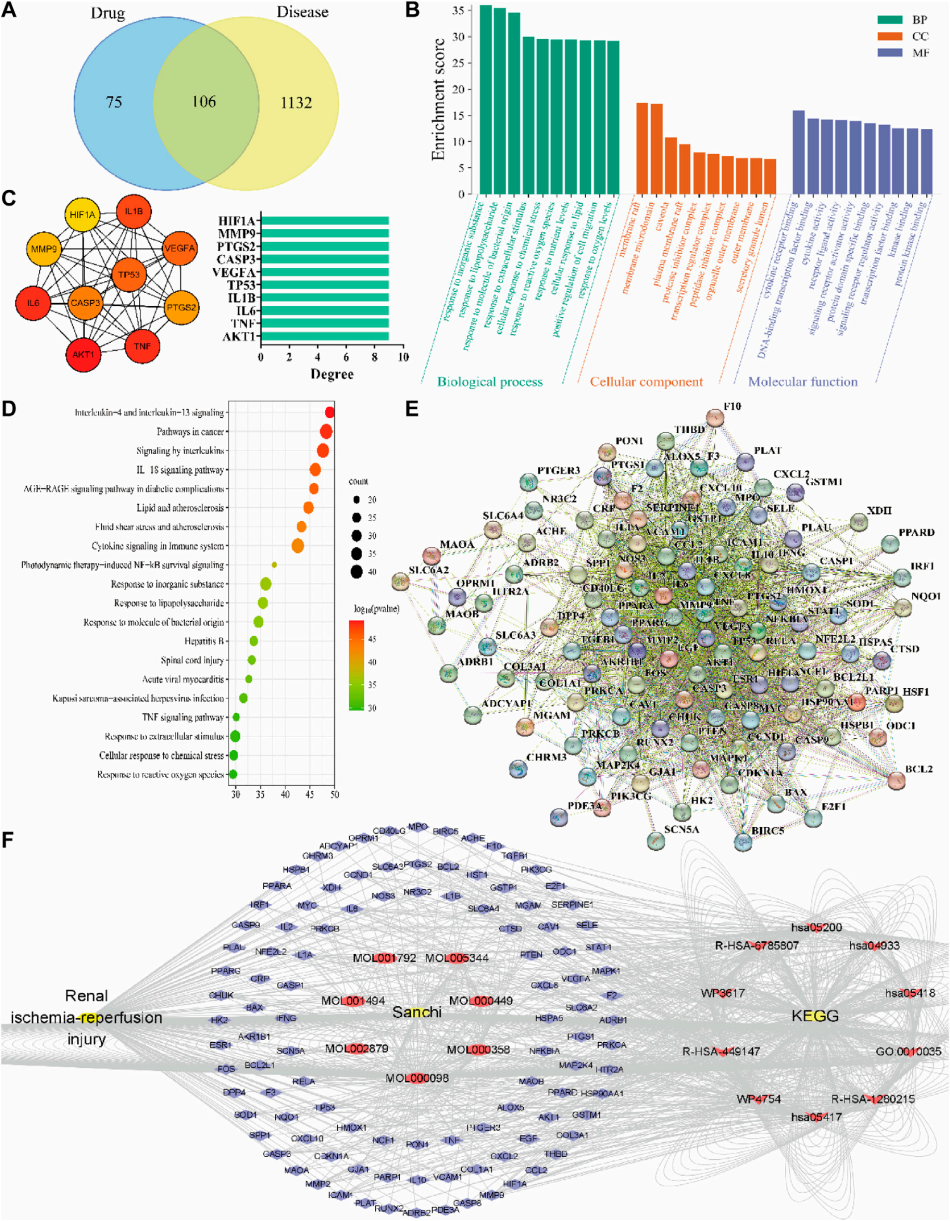
Results of drug-disease network relationship analysis using network pharmacology methods. **(A)** Drug-disease Venny intersection diagram, with the drug (Sanchi) targets on the left, the disease (RIRI) targets on the right, and the two intersection targets in the middle; **(B)** GO enrichment analysis of drug-disease; **(C)** Hub genes screened according to the PPI network relationships and rounded from the largest to smallest with the Degree value changes, and Degree–hub genes histogram with the Degree values as horizontal coordinates and genes as vertical coordinates; **(D)** KEGG signaling pathway analysis of drug-disease; **(E)** PPI network relationship diagram; **(F)** The network of “disease-drug active ingredient-key target-KEGG signaling pathway”, from left to right, is the disease (RIRI), drug (Sanchi) and its seven active ingredients Mol numbers, key targets (blue outer circle), and the first ten pathways of KEGG.

The results showed that 181 active ingredient targets of Sanchi were found in TCMSP, and 1,238 disease genes of RIRI were obtained in the GeneCards database. The relationship of gene targets between drug and disease was analyzed through the Venny 2.1.0 online platform, and 106 intersection genes (key targets) were obtained (Figure 3A). GO enrichment results of key targets were analyzed in the Metascape platform to - LogP values to screen the top 10 biological processes (BP), cellular components (CC), and molecular functions (MF). Among them, responding to inorganic substance is the most important BP, membrane raft is the most important CC, and cytokine receptor binding is the most important MF (Figure 3B). Meanwhile, KEGG signaling pathway analysis was performed for key targets, the top 20 signaling pathways were screened by - LogP values, and interleukin-4 and interleukin-13 signaling were the most important signaling pathways (Figure 3D).

The PPI network map was produced in the STRING network database platform using key targets (Figure 3E). The PPI relationship table was imported using the Cytoscape software, and the hub genes were screened from the largest to the smallest according to the Degree value: AKT1, TNF, IL-6, IL-1B, TP53, VEGFA, CASP3, PTGS2, MMP9, and HIF1A. Meanwhile, we constructed the hub gene network diagram and histogram (Figure 3C). Finally, a network relationship diagram of the disease, drug active ingredients, and key targets and KEGG signaling pathways was constructed (Figure 3F).

### 3.4 Molecular docking results

**FIGURE 4:**
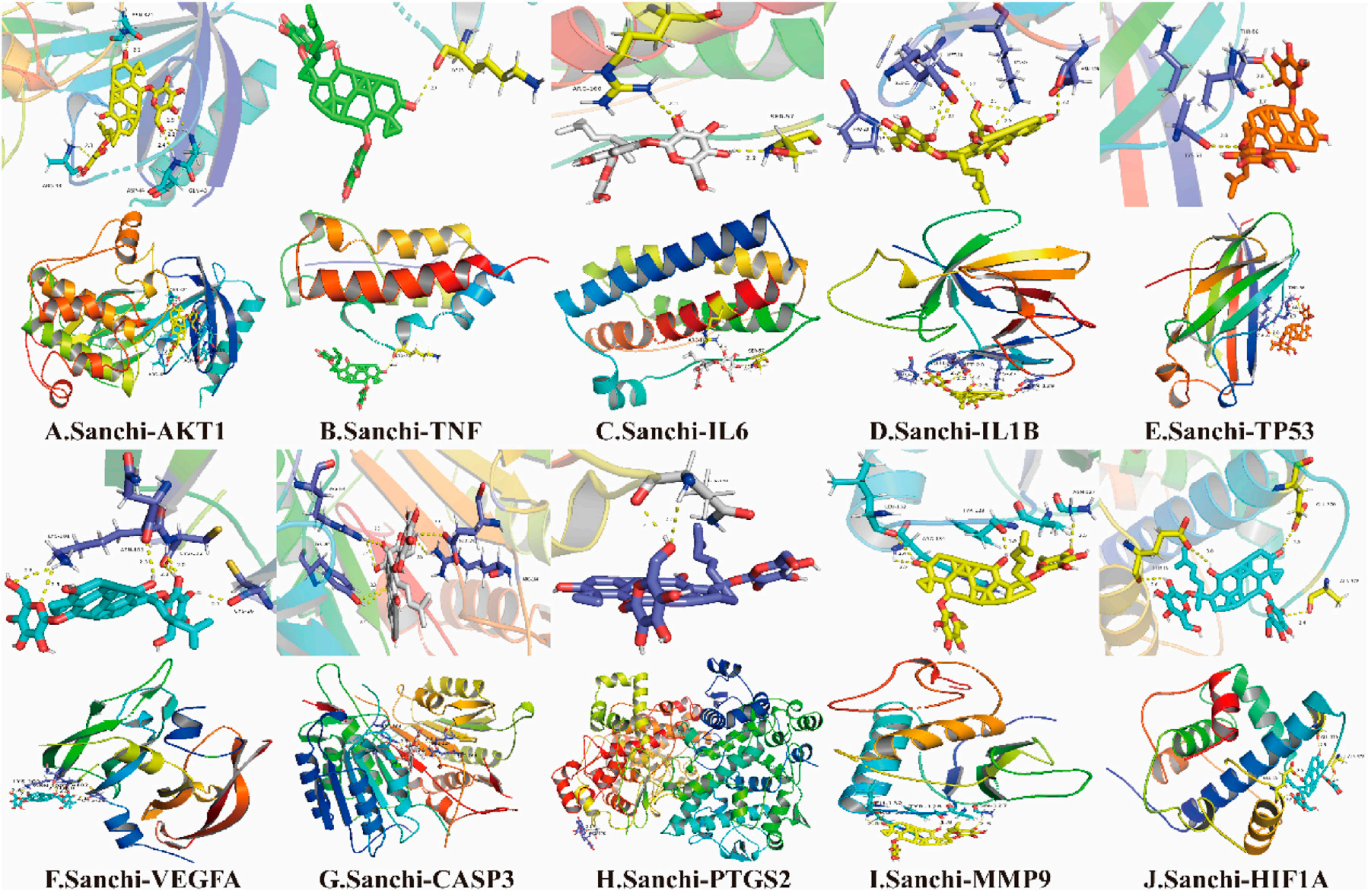
Molecular docking results of Sanchi and hub gene-related proteins.

When the binding energy is less than 0 kJ/mol, the drug ligand can spontaneously bind to the receptor protein, where less than -5 kJ/mol indicates good binding ability, and the lower the binding energy, the better the docking result (Gao et al., 2022). All hub genes were successfully molecularly docked according to the results, and the docking results were visualized using PyMOL software (Figure 4). Among them, MMP9 has the lowest binding energy to ligand molecules (-17.405 kJ/mol) and the strongest binding capacity (Table 2).

**TABLE 2:** Molecular docking results of Sanchi with hub genes.

Gene	PDB ID	Binding energy (kJ/mol)
AKT1	7NH4	−15.857
TNF	7MLR	−13.012
IL6	5ZO6	−13.012
IL1B	6Y8I	−13.347
TP53	7L1N	−7.740
VEGFA	6ZBR	−6.987
CASP3	7RN9	−14.937
PTGS2	5F19	−1.255
MMP9	4XCT	−17.405
HIF1A	7LVS	−14.477

### 3.5 qPCR results

**FIGURE 5:**
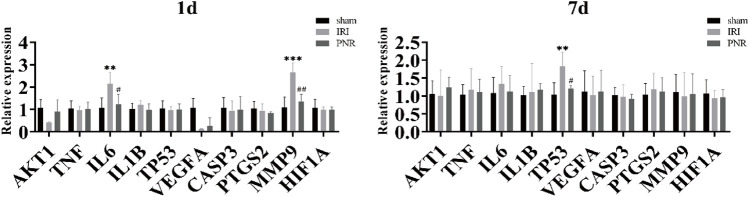
qPCR measurement of mRNA expression of ten hub genes in kidney tissues at days 1 and 7 postoperatively. Compared with the sham group, ***p < 0.001, **p < 0.01; compared with the IRI group, ^##^ *p* < 0.01, ^#^ *p* < 0.05.

The mRNA expression of ten hub genes (AKT1, TNF, IL-6, IL-1B, TP53, VEGFA, CASP3, PTGS2, MMP9, and HIF1A) was measured by qPCR in renal tissues at postoperative days 1 and 7, and the results showed that the *p* values of IL-6, MMP9, and TP53 genes were less than 0.05, with statistical significance. Compared with the sham group, the IRI group showed significantly elevated mRNA expression of IL-6 and MMP9 at day 1 postoperatively and TP53 at day 7 postoperatively. In contrast, the PNR group showed decreased mRNA expression of IL-6 and MMP9 at 24 h of reperfusion and TP53 at day 7 of reperfusion in IRI rats (Figure 5).

### 3.6 WB results

**FIGURE 6:**
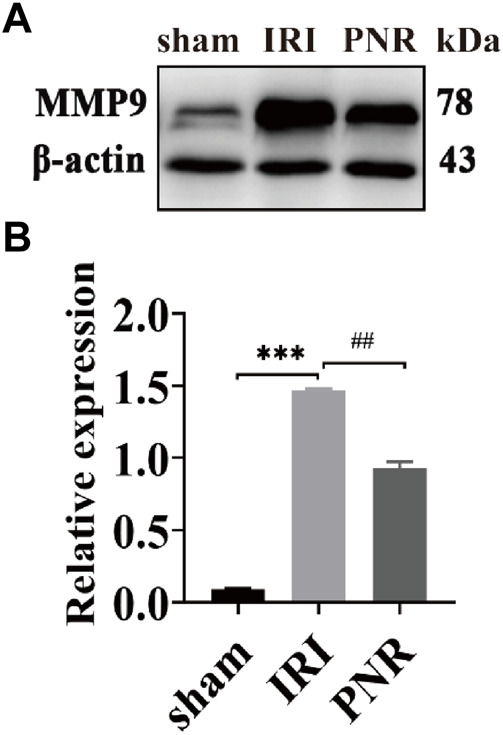
Results of MMP9 protein expression in kidney tissues at day 1 postoperatively (N = 3). **(A)** Protein bands for each group of MMP9 and *β*-actin detected by WB. **(B)** MMP9 protein expression in the kidneys of each group. Compared with the sham group, ***p < 0.001; compared with the IRI group, ^##^ *p* < 0.01.

According to the qPCR results, MMP9 expression at postoperative day 1 showed the most significant difference among the groups, and MMP9 was selected as a target for further validation of PNR action on RIRI. WB assay results showed increased MMP9 protein levels in the IRI and PNR groups compared to the sham group, but the PNR group had lower protein levels than the IRI group (Figure 6).

## 4 Discussion

In this study, data from the measurement of urine and blood biochemical indexes and observation of kidney morphology showed that the pretreatment with the PNR could reduce renal injury after IRI, improve TEC morphology, and inhibit apoptosis. Furthermore, the network pharmacological approach was used to elucidate the network relationship between Sanchi and RIRI. Then, we analyzed the results of GO enrichment and KEGG signaling pathways and used molecular docking to validate ten hub genes and analyze them by qPCR assay. These results indicated that PNR pretreatment could reduce the kidney injury caused by IRI in rats by inhibiting the expression of IL-6, MMP9, and TP53 in kidney tissues. In particular, MMP9 was further validated by WB assay for its important role in the pathological process of RIRI. Among them, the modulation of MMP9 and TP53 by the PNR in RIRI has not been reported, which provides a new research direction for the in-depth study of the mechanism of action of Sanchi on RIRI. In addition, these findings suggest that the PNR is an effective potential medicine for preventing RIRI.

### 4.1 PNR pretreatment has a protective effect against RIRI

By detecting the content of renal injury markers—creatinine and blood urea nitrogen, we found that PNR pretreatment significantly improved the abnormal Cr, SCr, and BUN content in the IRI group, reduced IRI-induced kidney damage in TTC staining, improved renal tubular cell morphology in HE staining, and reduced the number of positive cells in TUNEL staining. These results indicated that the PNR can improve renal injury and abnormal changes in TEC morphology caused by IRI and suggested that the PNR could play an important role in protecting the kidney of IRI rats.

The urine and blood biochemical index test is a common method for clinical examination of RIRI (Zhu et al., 2019). The results showed that IRI rats had elevated SCr and BUN levels in the blood and decreased Cr levels in the urine, suggesting kidney damage. Increased SCr and BUN content and impaired renal function after IRI were also observed in the study by Sun et al. (2020). In the observation of renal cell morphology, we could see that IRI rats pretreated with the PNR significantly reduced the expansion of TECs, infiltration of inflammatory factors in the interstitium, and tubular formation, and TEC injury is a key feature of the initial phase of RIRI (Basile et al., 2012). The study had shown that pectin-like polysaccharides extracted from Sanchi can improve TEC morphology and have an anti-fibrotic effect on the kidneys (Huang et al., 2022). In addition, PNR pretreatment reduced renal apoptosis in IRI rats, consistent with the findings that reduced apoptosis protected against renal injury (Shao et al., 2021), suggesting that apoptosis could be central in RIRI. In conclusion, the PNR can protect the kidney by affecting Cr, SCr, and BUN contents, improving renal cell morphology and reducing apoptosis, and can be used as a potential medicine to prevent the occurrence of RIRI.

### 4.2 Sanchi and RIRI network relationship

#### 4.2.1 Molecular mechanism of Sanchi and RIRI

In this experiment, through in-depth analyses of the network relationship between Sanchi and RIRI and its molecular mechanism, we identified the most important BP, CC, MF, and signaling pathways of Sanchi for treating RIRI, indicating that Sanchi can exert anti-inflammatory, antioxidant, immune regulatory, and other pharmacological effects on RIRI by using multiple targets and pathways, suggesting that Sanchi can be used to treat RIRI through multiple pathways, providing a certain research basis for the research of Sanchi in treating RIRI.

GO enrichment analysis revealed that the most important BP is response to inorganic substances, which is involved in apoptosis and mitochondrial dysfunction during RIRI (Hou et al., 2021). The most important CC is the membrane raft, which is involved in intracellular cholesterol transport, and during the renal injury phase, cholesterol transfer to the endoplasmic reticulum is increased and cholesterol ester production is increased. Cholesterol enrichment in the plasma membrane was shown to be protective against ischemic injury in the proximal tubule, as well as a manifestation of the response to injury (Zager et al., 2002). The most important MF is cytokine receptor binding, where cytokines bind to their receptors and play a gene-regulatory role in treating RIRI by mediating related signaling pathways (Ge et al., 2017).

The analysis of the first ten KEGG signaling pathways revealed that the first pathway was interleukin-4 and interleukin-13 signaling, and that the cytokines IL-4 and IL-13 mediated the polarization of renal macrophages or dendritic cells to the M2a (associated with wound healing and tissue repair) phenotype, thereby reversing recovery from RIRI (Zhang et al., 2017). The second pathway is pathways in cancer, signal transducer and activator of transcription 3 (STAT3) mediates many cancer-related signaling pathways and has an important role in immunomodulation against cancer and tumors (Zou et al., 2020), while it is also involved in promoting M2 polarization in renal macrophages, suppressing inflammatory responses, regulating immunity, and reducing RIRI (Chen et al., 2021; Liu et al., 2021d). The third pathway is signaling by interleukins; a variety of interleukins have been shown to regulate related genes with positive or negative effects on RIRI (Liang et al., 2017; Ding et al., 2019; Aiello et al., 2020). The fourth pathway is the IL-18 signaling pathway, showing that RIRI is associated with IL-18-induced pro-inflammatory factor production, and inhibition of IL-18 expression can effectively reduce renal injury and fibrosis (Yang et al., 2015; Liang et al., 2018). The fifth pathway is the AGE-RAGE signaling pathway in diabetic complications, and diabetic nephropathy is a diabetic microvascular complication and one of the causes of kidney injury. Activation of the AGE-RAGE signaling pathway will aggravate kidney injury through a series of pathways such as apoptosis, inflammatory response, oxidative stress, and mitochondrial dysfunction (Chen et al., 2020; Bhattamisra et al., 2021). The sixth pathway is lipid and atherosclerosis; lipid peroxidation occurs during RIRI, which is one of the important pathological processes of atherosclerosis, and anti-lipid peroxidation can reduce atherosclerosis (Erpicum et al., 2018; Bai et al., 2020). The seventh pathway is fluid shear stress and atherosclerosis, a chronic inflammatory disease of the vasculature driven by lipids and characterized by plaque formation at the site of pathogenesis (Schaftenaar et al., 2016); fluid shear stress can modulate vascular function to affect atherosclerosis formation (Cunningham and Gotlieb, 2005) and also reduce IRI by inhibiting apoptotic proteins (Zhang et al., 2021b). The eighth pathway is cytokine signaling in the immune system, where macrophages play an important role in the control of inflammation or repair after RIRI through T cells that contribute to the shift to an anti-inflammatory phenotype (Hasegawa et al., 2021). The ninth pathway is photodynamic therapy-induced NF-κB survival signaling, and inhibition of NF-κB signaling pathway-mediated inflammatory response and apoptosis protects against RIRI (Younis and Ghanim, 2022). The tenth pathway is response to inorganic substances; calcium overload is a key factor causing apoptosis of renal tubular cells after IRI (Hou et al., 2021), whereas H_2_O_2_ production increases during ischemia–reperfusion, and H_2_O_2_ affects renal mitochondrial function (Kamarauskaite et al., 2020).

In conclusion, Sanchi could reduce the damage of IRI to the kidneys by affecting response to inorganic substances, membrane raft, cytokine receptor binding, and the most important signaling pathways of interleukin-4 and interleukin-13 signaling.

#### 4.2.2 Validation of Sanchi and RIRI core genes

We established a PPI network relationship between Sanchi and RIRI intersection targets and screened ten hub genes for molecular docking and qPCR validation. It was found that the PNR could inhibit the expression of IL-6, MMP9, and TP53, and the WB assay further showed that the PNR could inhibit MMP9 protein expression. These results suggested that the PNR could regulate the related genes to reduce the kidney damage by RIRI and prompted us to pay attention to the changes in these related genes in the development of RIRI disease.

The PPI network relationship map was produced in the STRING network database platform, and the hub genes screened with the Degree value using the cytoHubba plug-in in Cytoscape software are AKT1, TNF, IL6, IL1B, TP53, VEGFA, CASP3, PTGS2, MMP9, and HIF1A, which may be involved in the molecular mechanism of Sanchi in treating RIRI (Chin et al., 2014; Zeng et al., 2021). Among them, the ten most closely related pairs of genes were obtained as AKT1–NOS3, AKT1–HSP90AA1, BCL2L1–TP53, CASP1–IL1B, CAV1–NOS3, CCND1–CDKN1A, CCND1–ESR1, CDKN1A–TP53, CHUK–NFKBIA, and ESR1–HSP90AA1. Analysis of these ten gene pairs showed that AKT1, CCND1, CDKN1A, ESR1, NOS3, HSP90AA1, and TP53 recurrently appear to be linked to other targets, which may play an important role in the treatment of RIRI by Sanchi (Murakami et al., 2017).

Using the screened hub genes and the drug ligand for molecular docking validation, the binding energy showed that both Sanchi and Hub genes were bound. Through qPCR detection of ten hub genes, we found that pretreatment of the PNR could regulate IL-6 and MMP9 at postoperative day 1 and TP53 at postoperative day 7 to protect the kidneys of IRI rats, which indicates IL-6, MMP9, and TP53 could be considered crucial molecular targets of the PNR in RIRI treatment. Furthermore, detection of MMP9 protein expression in renal tissues at postoperative day 1 by WB revealed that the PNR could effectively inhibit its expression, suggesting that we could pay attention to the effect of early RIRI on MMP9 and could prevent further attack of RIRI on the organism by inhibiting its expression.

IL-6 is a 26-kD secreted protein that stimulates antibody production by B cells and plays a role in physiological activities such as growth and development and regulation of immunity and metabolism and is also an important cytokine involved in inflammatory diseases of the body. Targeting the anti-IL-6 cascade may serve as an effective approach to treat inflammatory diseases (Kang et al., 2020). As a pro-inflammatory factor, IL-6 is an inflammatory marker of RIRI (Tian et al., 2020). Studies by Im, (2020) showed that ginsenosides Rb_1_, Rg_1_, and Rg_3_ can inhibit the expression of the inflammatory marker IL-6 and promote the regression of the inflammatory response. The application of notoginsenoside R_1_ pretreatment by Fan et al., (2020) reduced IL-6 levels in RIRI. It showed that the improvement of reperfusion 24-h kidney injury in this experiment was related to the anti-inflammatory effect of the pretreatment of the PNR, which reduced the expression of IL-6.

Matrix metalloproteinase 9 (MMP9) is a gelatinase of the zinc atom-dependent endopeptidase family of matrix metalloproteinases (MMPs) (Xin et al., 2015). MMP9 can disrupt the vascular basement membrane to reduce the renal vascular density and digest the extracellular matrix (ECM) to facilitate the spread of monocyte infiltration, and indirectly activate neutrophils and promote inflammatory responses (Kwiatkowska et al., 2016). The upregulation of MMP9 expression is associated with many kidney diseases (Ersan et al., 2017), and the degree and duration of its expression could affect the extent of kidney injury (Novak et al., 2010). Notoginsenoside R_1_ could improve ischemic injury by downregulating the MMP9 expression through the Caveolin1/MMP2/9 pathway (Liu et al., 2021a). PNGL (notoginseng leaf triterpene) reduced the pro-inflammatory mediator MMP9 and inhibited MAPK and NF-κB signaling pathways, which had a protective effect against IRI (Xie et al., 2019). In our observation, the expression of MMP9 increased in RIRI but decreased in the PNR group, which indicated that the inhibition of MMP9 expression at 24 h of reperfusion by pretreatment of the PNR prevented further damage to the kidney tissue by ischemia–reperfusion.

The tumor suppressor gene TP53 can encode p53 protein. As the main transcription factor of the organism, p53 protein is involved in the transcription of a large number of protein-coding genes, which can mediate transcriptional regulation of biological life activity processes and play a key role in regulating cell cycle, apoptosis, cell autophagy, DNA repair, cellular senescence, and other cellular metabolism processes (Aubrey et al., 2016; Hu et al., 2019). p53 is involved in renal tubular cell senescence and accelerates renal fibrosis (Luo et al., 2018). Knockout of p53 can reduce kidney cell apoptosis, cell necrosis, oxidative stress, inflammatory response, fibrosis, and other factors to improve RIRI (Ying et al., 2014). Thus, pretreatment of the PNR inhibited TP53 gene expression to reduce renal injury at day 7 of reperfusion.

In summary, using network pharmacology methods, molecular docking techniques, qPCR, and WB, we investigated the molecular mechanism of the PNR to improve RIRI, indicating that IL-6, MMP9, and TP53 have important roles in inflammatory responses, and also affect pathophysiological activities such as apoptosis, cell necrosis, and oxidative stress in the organism. Moreover, this experiment found for the first time that the PNR could inhibit MMP9 and TP53 expression to prevent further kidney damage after IRI, which provided some theoretical basis for the subsequent clinical application of the PNR.

## 5 Conclusion

The present results showed that pretreatment with the PNR improved effectively renal injury, reduced apoptosis in RIRI rats, and protected ischemic–reperfused kidneys. The main mechanism may be related to the regulation of IL-6, MMP9, and TP53 genes. Therefore, PNR pretreatment may be a potential strategy for treating RIRI.

## Data Availability

The original contributions presented in the study are included in the article/Supplementary Material, further inquiries can be directed to the corresponding authors.
