# Supplementary material for: A molecular network-based pharmacological study on the protective effect of Panax notoginseng rhizomes against renal ischemia–reperfusion injury
Source: Front Pharmacol. 2023 Apr 18;14:1134408. doi: 10.3389/fphar.2023.1134408 (PMC10151715; doi:10.3389/fphar.2023.1134408)
Supplement: Supplementary file 1 [file Table1.DOCX]

Please click the links below to view the specific raw data.

1. Layout picture:

<https://www.jianguoyun.com/p/DVqEva4Q06KmCxitse8EIAA> (访问密码：1234)

2. Network pharmacology

<https://www.jianguoyun.com/p/DVdcU5AQ06KmCxjIse8EIAA> (访问密码：1234)

3. Pharmacological experiment

<https://www.jianguoyun.com/p/DWsZLvIQ06KmCxjHsO8EIAA> (访问密码：1234)
